# Medical Attendance of Londoners

**Published:** 1903-07-18

**Authors:** 


### HOSPITAL ADMINISTRATION.

### CONSTRUCTION AND ECONOMICS.

### MEDICAL ATTENDANCE OF LONDONERS.

If ever the history of British self-reliance and individual effort should come to be written, as it is the fashion to write histories of abstract ideas, the chapter upon voluntary organisation for the relief of the sick poor should be one of the most important. It would be a concrete history of zealous individuals striving always in a private capacity to keep abreast of the rapidly growing needs of medical charity under increasingly complex social conditions. That the effort has been on the whole successful is apparent from the fact, which we take from a recent speech by Sir Henry Burdett, reprinted as a pamphlet,* that the total expenditure for 1902 of all London medical institutions whose names are on the Sunday Fund list was £1,161,133, and that their income was £1,158,000, leaving a deficiency on the year of about £3,100 only. The grand object of making ends meet has, at all events, been attained hitherto, so that the speaker whom we have quoted says:—"I claim that to day the voluntary hospitals are for all practical purposes financially secure." But the balance sheet is not everything. Although there is not a shadow of suspicion upon its correctness, and there is therefore every such assurance as may be derived from solvency in favour of moral soundness, yet there is a great deal of dissatisfaction with the existing state of things. Even the dissatisfaction is a healthy sign. We should not be British if we did not grumble; and if we did not look out in time we should sink in apathy and be overwhelmed in confusion.

The occasion of the comprehensive speech which gives rise to our comments was the annual meeting of a small dispensary in Marylebone; and the occasion was perfectly well fitted for a review of the situation. The history of dispensaries lies at the beginning of the history of voluntary combinations for the relief of the sick. It also starts the history of the eternal conflict between the interests of consultants and general practitioners, inasmuch as the very first dispensary, opened in Warwick Lane in 1689, was the enterprise of the College of Physicians, and was thwarted from the outset, and at length obliged to put up its shutters, by the opposition of Apothecaries' Hall. The next effort opens a new chapter in the history. It shows us the enthusiasm of Dr. Lettsom, some two generations after, working single-handed from the Aldersgate Dispensary amongst the crowded courts and debtors' prisons. He appears to have met with no professional opposition, because no one supposed that Lettsom had a personal end in view, or that he was all the while seeking to build up a consultant's practice. But it is only in the early days of charities that we meet with such enthusiasms. They become inevitably organised, a board meets regularly in the way of business, there is a paid secretary, and medical candidates come forward eager for the privilege of serving the charity gratuitously. Still, it is the original dispensaries, as Sir Henry Burdett heartily grants them, that have kept the spirit of medical charity in its purest form. They are doing good work at small cost; they are a kindly and unobtrusive influence in their respective neighbourhoods; and when they have been organised on a greater scale, as at Battersea, they have proved to be at once the most popular and the least aggressive upon the interests of the private practitioners.

It will be impossible, in our space, to summarise the frequent criticisms and practical suggestions of the speech referred to, but we notice one or two leading themes. The great hospitals of London are attended by two things, each of which is really an adjunct, accessory, or accretion upon the original charity—or upon the original idea of the charity—an out-patient department and a medical school. The growth of the out-patient department is comparatively recent. One can easily go back to the time when certain of the great hospitals had only one assistant surgeon, and perhaps no assistant physician. The growth of the medical school can be followed almost within the lifetime of the late Sir James Paget, whose own enthusiasm was, indeed, the first occasion of the appointment of a demonstrator or lecturer on pathological anatomy. Paget used to prælect spontaneously upon the specimens, and the pupils of the hospital, who gathered round him, petitioned for his formal appointment. It might have been well if, in those early days, some step had been taken to found a central school for all but clinical teaching, like the École de Médecine. Whatever defects there were at the start have followed the rule of congenital defects in the body, to grow with its growth and strengthen with its strength. Hence the anomaly, so patent to foreign observers, of twelve professors of anatomy, twelve professors of physiology, twelve Virchows, twelve Christisons, and everything else by the dozen. Sir Henry Burdett protests strongly against the practice, at certain of the hospitals, of diverting a part of the voluntary contributions for the support of the medical school. Equally emphatic is his condemnation of the expensiveness and abuse of the out-patient departments, and of minor incidental grievances arising out of their crowded state. He sees in the future a "reformed out-patient department" in the shape of a provident dispensary at each of the hospitals, to which the patient would be handed over after being seen, and approved, by one of the hospital staff. The abuse of the in-patient department is, he thinks, less; the admission of those not actually poor having become inevitable for various reasons, which have been well pointed out by Sir Frederick Treves. But there ought to be some regular means of enabling the better sort to pay; and such a scheme the reader will find elaborated in the last two pages of the pamphlet which we have been reviewing.

* "The Medical Attendance of Londoners: an Economical System, etc." A speech made at the annual meeting of the Portland Town Free Dispensary by Sir Henry Burdett, K.C.B. London, 1903.

